# Process and Microstructure to Achieve Ultra-high Dielectric Constant in Ceramic-Polymer Composites

**DOI:** 10.1038/srep35763

**Published:** 2016-10-21

**Authors:** Lin Zhang, Xiaobing Shan, Patrick Bass, Yang Tong, Terry D. Rolin, Curtis W. Hill, Jeffrey C. Brewer, Dennis S. Tucker, Z.-Y. Cheng

**Affiliations:** 1 Materials Research and Education Center, Auburn University, Auburn, AL 36849, USA; 2 Marshall Space Flight Center, Huntsville, AL 35812, USA

## Abstract

Influences of process conditions on microstructure and dielectric properties of ceramic-polymer composites are systematically studied using CaCu_3_Ti_4_O_12_ (CCTO) as filler and P(VDF-TrFE) 55/45 mol.% copolymer as the matrix by combining solution-cast and hot-pressing processes. It is found that the dielectric constant of the composites can be significantly enhanced–up to about 10 times – by using proper processing conditions. The dielectric constant of the composites can reach more than 1,000 over a wide temperature range with a low loss (tan *δ* ~ 10^−1^). It is concluded that besides the dense structure of composites, the uniform distribution of the CCTO particles in the matrix plays a key role on the dielectric enhancement. Due to the influence of the CCTO on the microstructure of the polymer matrix, the composites exhibit a weaker temperature dependence of the dielectric constant than the polymer matrix. Based on the results, it is also found that the loss of the composites at low temperatures, including room temperature, is determined by the real dielectric relaxation processes including the relaxation process induced by the mixing.

Flexible dielectrics with a high dielectric constant are critical for many applications, such as energy-storage capacitors, gate dielectrics in field-effect transistors, and pulse-power and system-on-package technologies^1^,^2^. Although polymers are flexible, they exhibit a low dielectric constant^3^. To create flexible dielectrics with a high dielectric constant, a great deal of effort has been devoted in recent years to the development of polymer-based dielectric composites, especially the 0–3 composites in which a dielectric polymer matrix is filled with particles of either conductors or dielectrics^3^,^4^,^5^,^6^,^7^.

The dielectric response of a dielectric polymer filled with conductive particles – conductor-dielectric composite (CDC) – is determined by a percolation phenomenon. The CDCs are insulative/dielectric when the filler content (*φ*) is less than a certain value (the percolation threshold *φ*_*c*_). In the insulative regime, the dielectric constant of a CDC increases with increasing the *φ*. As the *φ* approaches the *φ*_*c*_, the dielectric constant of the CDC can reach a very high value^4^,^5^,^6^. Therefore, the composites using different conducting fillers (i.e. metal, carbon, carbon nanotube, conducting polymer) in different shapes (i.e. spherical particles^8^,^9^,^10^, fibers/rods/wires^11^,^12^, plates/discs^13^) have been studied^4^,^5^,^6^,^14^. When *φ* is close to *φ*_*c*_, the CDCs can exhibit a high dielectric constant (~1,000), which is, however, usually associated with a high dielectric loss (tan *δ* ~ 10^0^ to 10^2^)^5^.

The dielectric response of a dielectric polymer filled with dielectric particles – dielectric-dielectric composite (DDC) – is determined by many factors, such as the dielectric properties of each constituent, size and shape of the filler particles, and the composition (i.e. the *φ*) and the microstructure (i.e. the distribution and connectivity of the filler particles) of the composites^3^,^4^,^5^,^6^. For the development of DDCs, dielectric ceramics, especially ferroelectric/relaxor ceramics such as BaTiO_3_, Pb(Mg_1/3_Nb_2/3_)O_3_, Pb(Zr,Ti)O_3_, (Ba,Sr)TiO_3_ have been widely used as fillers due to the fact that these ceramics exhibit a high dielectric constant (10^3^ ~ 10^4^)^15^,^16^,^17^,^18^,^19^. However, the dielectric constant of these DDCs, even with the *φ* reaching 50 vol.%, is barely higher than 100 (mostly less than 50)^5^. Additionally, the dielectric properties of ferroelectrics/relaxor ceramics are strongly dependent on the temperature and exhibit a strong piezoelectric effect^20^,^21^, which are undesirable for dielectric applications. The recent study on CaCu_3_Ti_4_O_12_ (CCTO) provides a new opportunity to develop high-performance DDCs because CCTO ceramic exhibits a giant dielectric constant (>10^4^) with a weak temperature dependence over a wide temperature range and does not have the piezoelectric effect^22^,^23^,^24^. It was reported that CCTO-P(VDF-TrFE) composites with a *φ* of 50 vol.% at room temperature exhibit a dielectric constant of ~400 at 100 Hz with a dielectric loss of about 0.6^25^. The dielectric constant observed in the CCTO-P(VDF-TrFE) is significantly higher than that observed in other DDCs. Since the report, CCTO-polymer composites become an interesting topic and have been studied by many research groups^26^,^27^,^28^,^29^,^30^,^31^,^32^,^33^,^34^,^35^,^36^,^37^,^38^,^39^,^40^. CCTO particles, ranging in size from nanometers to micrometers, have been used in the studies. The dielectric constant of most CCTO-polymer composites is about or less than 100, while some reports show a significantly high dielectric constant^26^,^27^,^28^,^29^,^30^,^31^. For example, in a study of CCTO-PVDF composites using CCTO prepared by a wet chemical process, it was reported that the composites using nano-sized CCTO exhibited a dielectric constant of >10^6^ at 100 Hz at room temperature, but with an extremely high loss (tan *δ* ~ 50)^33^. Regarding the size of CCTO, some reports show that the composites using micro-sized CCTO exhibited a higher dielectric constant^35^, while some show that the composites using nano-sized CCTO exhibit a higher dielectric constant^33^.

The huge differences in the dielectric properties observed in the CCTO-polymer composites may originate from: 1) the dielectric properties of CCTO; 2) the microstructure of the composites. It is well known that the dielectric properties of CCTO itself are very sensitive to the preparation process and to the impurities in the CCTO^22^,^23^,^24^. Therefore, the CCTO prepared by using a wet chemical process and the CCTO prepared by using a traditional ceramic process can be very different in terms of the dielectric properties.

The microstructure of the composites plays an important role on their dielectric properties. For example, in extreme cases for a binary system, two constitutes can be connected either in parallel or in series, which would result in a huge difference in the dielectric response of the composites. As described by the widely accepted Wiener limit^5^,^41^, for the composites with the same composition but different connectivities, a real composite exhibits a smaller dielectric constant than the composite with the parallel connection, but a higher dielectric constant than the composites with the series connection; for composites with the same microstructure/connectivity but different compositions, the dielectric constant of the composites changes with the composition monotonically. For example, if a dielectric polymer is filled with a dielectric with a higher dielectric constant, the dielectric constant of the composites would monotonically increase with increasing the content of filler. The microstructure of a composite is determined by its preparation process. Most reported composites are prepared using a one-step process, such as solution cast, spin-coating, hot mold, etc.^5^.

In this paper, the process used to prepare the composites is studied to control the microstructure, so the dielectric response of a composite can be manipulated. Here, a solution cast is combined with a hot-pressing process to prepare the composites. The composite film is first prepared using the solution casting process. Then, the hot-pressing process is used to prepare the final composites. During the hot-pressing process, multiple layers of the as-cast films with the same composition are stacked together as the starting materials. It is found that, by using controlled hot-pressing conditions, the dielectric constant of the composites can be significantly enhanced. For example, at room temperature, the dielectric constant at 100 Hz for the composites with 50 vol.% CCTO can be enhanced from about 80 for the solution-cast films to about 760 for hot-press composites; at 75 °C, the composites exhibit a dielectric constant of more than 1,300, which is considerably higher than the dielectric constant reported for the DDCs^5^. The dielectric constant of the DDCs reported here is similar to that of the CDCs with a composition close to the *φ*_*c*_, but these DDCs have a much smaller dielectric loss (tan δ ~ 10^−1^) compared to the CDCs (tan δ ~ 10^0^ to 10^2^).

## Results and Discussion

### Dielectric properties of solution cast CCTO-P(VDF-TrFE) composites

**Figure 1:**
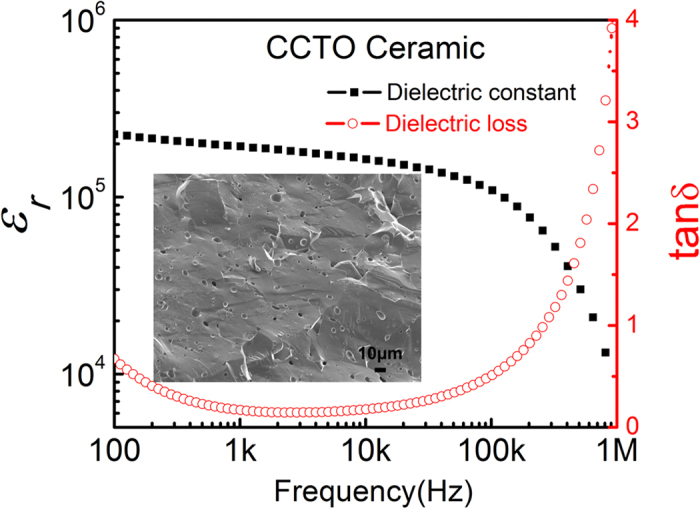
Frequency dependence of the dielectric constant and loss for CCTO ceramics at room temperature (Insert: SEM micrograph of CCTO ceramic cross section).

The dielectric properties of CCTO ceramics at room temperature are presented in Fig. 1, where the SEM micrograph of the cross section is also shown. Clearly, the ceramics exhibit a high dielectric constant. For example, the dielectric constant at 1 kHz is about 190,000 with a loss of 0.17. It is also noticed that there is a relaxation process around 1 MHz. Due to this relaxation process, the dielectric constant of the ceramics at high frequency is low: the dielectric constant at 1 MHz is about 10,000. Micro-sized CCTO particles prepared from the ceramics were used as the filler to prepare the composites. P(VDF-TrFE) 55/45 mol.% copolymer was selected as the polymer matrix since it has a high dielectric constant and weak piezoelectric effect.

**Figure 2:**
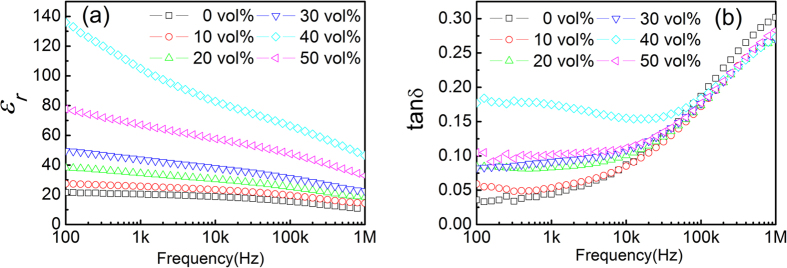
Frequency dependence of the dielectric properties for solution cast CCTO-P(VDF-TrFE) composites with 0 to 50 vol.% of CCTO at room temperature: (**a**) dielectric constant, and (**b**) dielectric loss.

The dielectric properties of the CCTO-P(VDF-TrFE) solution-cast composites at room temperature are shown Fig. 2. As the CCTO content (*φ*) increases, the dielectric constant of the composites increases slowly for the *φ* ranging from 0 to 30 vol.%, and then rapidly as *φ* increases from 30 to 40 vol.%. However, it is found that composites with *φ* = 50 vol.% exhibit a lower dielectric constant than composites with *φ* = 40 vol.%. That is, the composites with 40 vol.% CCTO exhibit the highest dielectric constant: the dielectric constant at 100 Hz is more than 130 with a loss less than 0.2, which can be seen as typical dielectric properties of DDCs with a high dielectric constant^5^.

Regarding the dielectric loss, it is found that the frequency can be divided into two regimes: low frequency (<100 kHz) and high frequency (>100 kHz) regime. In the low frequency regime, the dielectric loss increases with increasing *φ* from 0 to 40 vol.%. However, the composites with 50 vol.% CCTO exhibit a lower loss than the composites with 40 vol.% CCTO. In the high frequency regime, the loss of the composites is almost independent of *φ*, but is strongly dependent on the frequency as shown in Fig. 2(b). Interestingly, the composites exhibit a lower loss than the polymer matrix.

### Hot-press composites using multilayer structures

Due to the poor wettability, it was reported that the solution cast CCTO-P(VDF-TrFE) composites do not have a uniform microstructure. For example, a thin polymer layer can be observed on the bottom side of the as-cast film^25^. It was reported that the hot-pressing process can be used to improve the composites^25^. Here a systemic study on the hot-pressing condition is carried out. For the multilayer stack used in the hot-pressing process, both the number of layers and the configuration/pattern of the stacked forms are studied along with the hot-pressing time. Regarding the configuration of the stack, the as-cast composite films were labeled as “P” for the bottom side with a thin polymer layer and “C” for the top side. The multilayer stacks had two different configurations: (a) PCPC pattern, in which the “P” side of an as-cast film was placed in contact with the “C” side of the next as-cast film; (b) PCCP pattern, in which the “P” side of one as-cast film is in contact with the “P” side of the next as-cast film.

**Figure 3:**
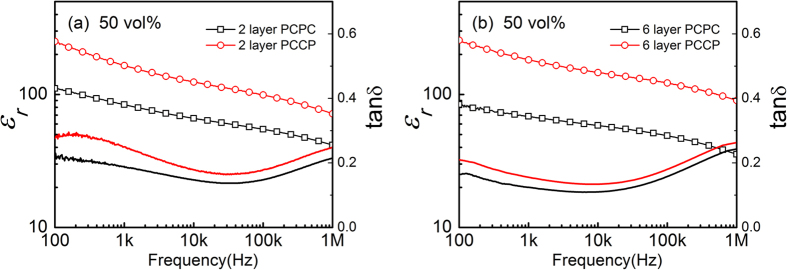
Frequency dependence of the dielectric properties of CCTO-P(VDF-TrFE) composites with 50 vol.% of CCTO with different hot-press conditions: (**a**) 2 layers with PCPC and PCCP configurations for 10s, and (**b**) 6 layers with PCPC and PCCP configurations for 10s.

**Figure 4:**
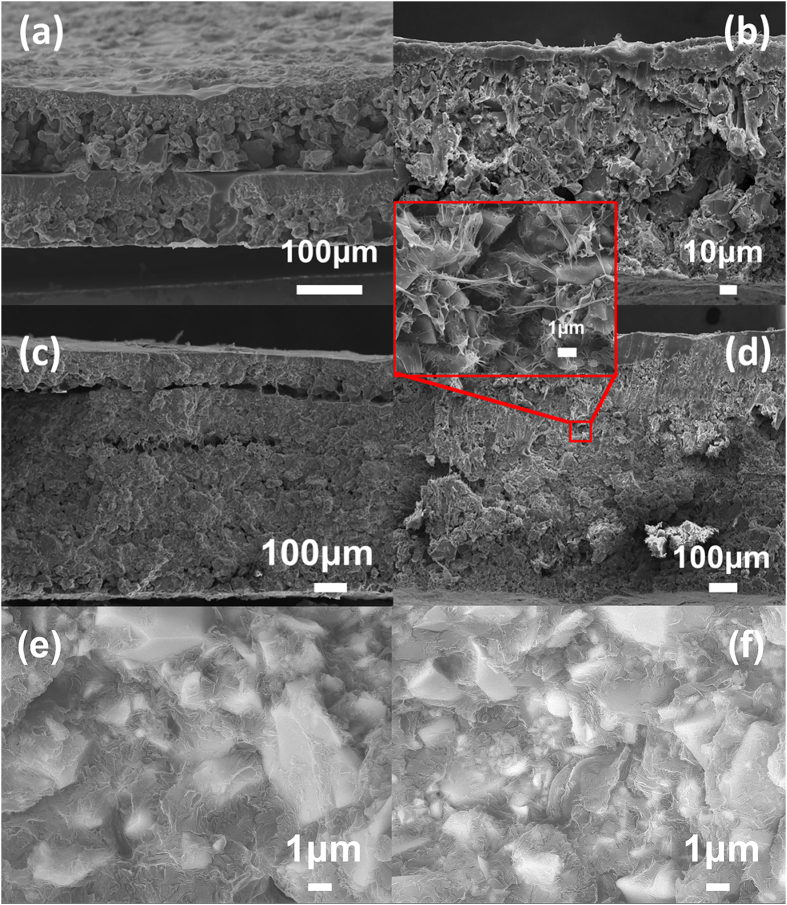
SEM of fresh, fractured cross-section of different CCTO-P(VDF-TrFE) composites prepared under different hot-pressing conditions: (**a**) 50 vol.%, 2 layers with a PCPC pattern 10s, (**b**) 50 vol.%, 2 layers with a PCCP pattern 10s, (**c**) 50 vol.%, 6 layers with a PCPC pattern 10s, (**d**) 50 vol.%, 6 layers with a PCCP pattern 10s, (**e**) 40 vol.%, 4 layers with a PCCP pattern 20s, (**f**) 50 vol.%, 6 layers with a PCCP pattern 30s.

Figure 3 shows the dielectric properties of two groups (2 layers and 6 layers) of composites at room temperature, where the results from composites with 50 vol.% CCTO prepared using two configurations (i.e. PCPC and PCCP) are presented. Comparing with the results of the solution-cast composites shown in Fig. 2, the hot-press composites exhibit a higher dielectric constant. More interestingly, it is found that the hot-press composites prepared using the PCCP pattern/configuration exhibit a significantly (2 to 3 times) higher dielectric constant than composites using the PCPC pattern, while the dielectric loss of the composites prepared using the PCCP pattern are only slightly higher than that of the PCPC composites. This indicates that the dielectric properties of the composites can be significantly improved by using the PCCP pattern during the hot-pressing process. The microstructure of the composites was examined using SEM as shown in Fig. 4. Fig. 4(a-d) are composites with 50 vol.% CCTO prepared with a hot-pressing time of 10 s, but with different numbers of layers, are presented. One can find that the composites prepared using the PCCP pattern, Fig. 4(b,d), have a more uniform and denser microstructure than the composites prepared using the PCPC pattern, Fig. 4(a,c). The SEM images shown in Fig. 4(a–d) indicate the overall uniformity of the composites. In order to show the interface between the filler and matrix, the SEM images with a higher resolution are shown in Fig. 4(e–f), which clearly indicates there are good connections between the filler particles and the matrix in the hot-press composites. These images demonstrate the effectiveness of the hot pressing process in improving the connections between the filler particles and matrix. Therefore, the high dielectric constant observed in the composites prepared using the PCCP pattern can be linked to the dense and uniform microstructure of the composites.

**Figure 5:**
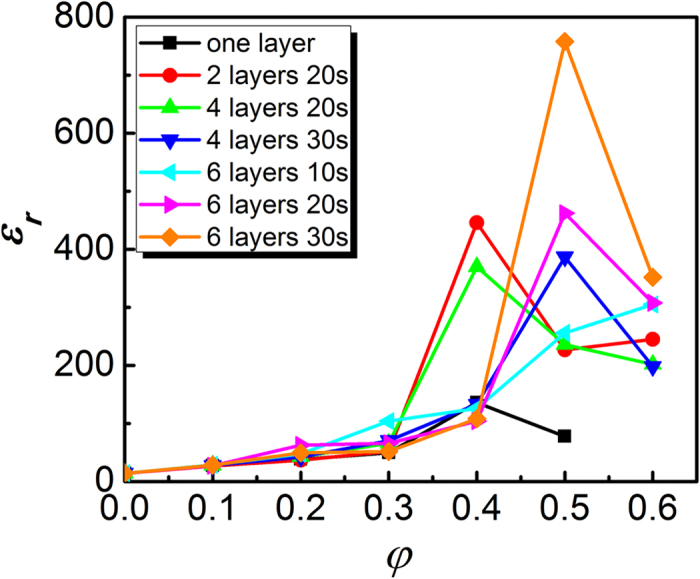
Dependence of dielectric constant at 100 Hz on CCTO content for the composites at room temperature. The composites were prepared under different hot-pressing conditions: 1, 2, 4, and 6 layers with a PCCP configuration for 10, 20, and 30 s.

The PCCP pattern was further studied for the composites with different compositions. Both the number of layers used in the hot-pressing process and the hot-pressing time were studied. The results are summarized in Fig. 5, where the composites prepared using 2, 4, and 6 layers with different hot-pressing times are presented. Regarding hot-pressing time, if too short, the particles are not able to move to form a new distribution, but if too long (>40 second), there are resulting negative changes in the polymer. Therefore, three hot-pressing times (10, 20 and 30 second) were used in the experiments. To clearly show the trend, the results are summarized in Table 1.

The data shown in Fig. 5 clearly indicates that under each hot-pressing condition, the dielectric constant of the composites increases initially, and then decreases with increasing the *φ*, which is similar with what is observed in Fig. 2 for the solution-cast composites. This results in the appearance of a maximum dielectric constant for composites with the same composition but different processing conditions. For example, for composites prepared using two and four layers with a hot-pressing time of 20 s, the composites with 40 vol.% CCTO exhibit the highest dielectric constant; for composites prepared using four layers with 30 s and using six layers with 20 and 30 s, the composites with 50 vol.% CCTO exhibit the highest dielectric constant. The highest dielectric constant (>750 at 100 Hz) obtained here is well higher than the dielectric constant obtained in other DDCs. This high dielectric constant is comparable to the dielectric constant obtained in the CDCs with the *φ* close to *φ*_*c*_, but the composites reported here have a much smaller loss (tan*δ* ~ 0.4 at 100 Hz) compared to the CDCs.

The change in the dielectric properties of the composites with the hot-pressing condition, shown in Fig. 5, is certainly related to the change in the composites during the hot-pressing process. Considering the temperature used in the hot-pressing process, one would not expect any change in the ceramics. The only changes that can be induced by the hot-pressing process are: (1) the microstructure of the polymer matrix (i.e. crystallinity, size and shape of the crystalline regions, structure of the crystal, and the conformation of the polymer chains in the amorphous region); and (2) the microstructure of the composite. It is known that the change in the dielectric constant of the copolymer used here by changing its microstructure is small at room temperature. For example, the dielectric properties of the copolymer prepared under different conditions are almost the same. Therefore, one can conclude that the observed significant change in the dielectric constant by the hot-pressing process originates from changes in the microstructure of the composites during the process.

**Figure 6:**
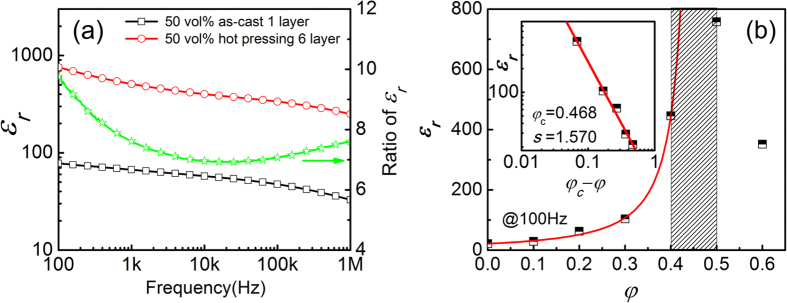
(**a**) Frequency dependence of the dielectric constant for composites with 50 vol.% CCTO prepared under different conditions: solution-cast composites and hot-pressing using 6 layer with a PCCP configuration for 30 s. (**b**) The dielectric constant versus the composition for the composites that exhibit the highest dielectric constant for each composition. Insert figure shows the fitting curve by Eq. (1).

As revealed by the SEM observation shown in Fig. 4, during the hot-pressing process, there are two types of possible changes in the microstructure: (1) making the composites denser by eliminating porosity in the as-cast films and by improving connections between filler particles and matrix; (2) changing the distribution of the filler particles in the matrix. To further illustrate the influence of the hot-pressing process on the dielectric response of the composites, the frequency dependence of the dielectric constant is plotted in Fig. 6(a) for composites with 50 vol.% CCTO, where the hot-pressing composites were prepared using six layers and 30s. One can find that the ratios of the dielectric constant obtained in the composites after hot-pressing to the dielectric constant of the solution-cast composites are about 7~10. That is, by using the hot-pressing process with the proposed conditions, the dielectric constant is improved by 7~10 times. Interestingly, it is found that this ratio is not independent of frequency. If the only effect of the hot-pressing process is eliminating the pores in the as-cast film and/or improving connections between the filler particles and matrix, it would be expected that this ratio is almost independent of the frequency. Therefore, the frequency dependence of this ratio indicates that in addition to eliminating porosity and improving connections between the filler particles and matrix, the distribution of the filler particles in the matrix is changed during the hot-pressing process.

It is known that the dielectric relaxation processes in a composite can originate from the relaxation processes existing in each constituent and new dielectric relaxation processes are introduced by the mixing of two different dielectrics, such as the Maxwell-Wagner effect^42^. The change in the distribution of the filler particles in a composite would not affect the relaxation processes of each constituent, but would have a stronger influence on the new dielectric relaxation processes arising from mixing. Comparing the uniform and non-uniform distribution of the filler particles within a matrix, a non-uniform distribution results in a weaker mixing effect since there are more filler particles directly connected to each other. Therefore, one would expect that the new relaxation process has the strongest contribution to the dielectric properties of the composites when the filler particles are uniformly distributed in the matrix. Based on this, one can speculate that during the hot-press process, the distribution of the CCTO particles changes from non-uniform in the as-cast films to a uniform distribution, which results in the highest enhancement on the dielectric constant of the composites.

Based on the above discussion, one can assume that the composites with the highest dielectric constant at each composition have a similar microstructure (i.e. dense and uniform). It is well known that the microstructure uniformity of a composite has a strong influence on the measured dielectric constant of the samples of the composite^10^. If a composite has a uniform microstructure, the dielectric constant observed from different pieces of the composite should be the same. If a composite has a non-uniform microstructure, the dielectric constant observed from different pieces of the composite would be different. For the composites with less than 40 vol.% CCTO, it is experimentally found that the relative error in the measured dielectric constant is less than 5%. For the composites with 40 and 50 vol.% CCTO, the relative error changes over a very big range, from up to 30% down to a few percent. This is consistent with the data shown in Fig. 5: the hot pressing process has a weaker influence on the dielectric constant of the composites with lower CCTO content, but a very strong impact on the dielectric constant of the composites with 40 and 50 vol.% CCTO. More interestingly, a strong correlation between the dielectric constant and relative error is observed. The higher the dielectric constant, the smaller the relative error. In other words, for the composites with the same CCTO content but prepared using different hot pressing processes, the composite with the highest dielectric constant shows the smallest relative error (i.e. less than 10%). This is consistent with the conclusion made above that the composite with a uniform microstructure exhibits a higher dielectric constant.

For the composites with the same CCTO content, the composite with the highest dielectric constant is selected to represent the composite with a uniform microstructure. The composition dependence of the dielectric constant for these composites is plotted in Fig. 6(b), where the dielectric constant at 100 Hz is used. Again, it is found that the dielectric constant of the composites increases initially, and then decreases, with increasing the *φ*.

Regarding the composition dependence of the dielectric constant for the composites, there are many models/equations introduced for DDCs^5^,^41^. However, none of these models/equations would be able to fit the data shown in Fig. 6(b) since the dielectric constant decreases with increasing *φ* at higher *φ*. However, the data shown in Fig. 6(b) is very similar to what is obtained in the CDCs, where the dielectric constant increases (*φ* < *φ*_*c*_), and then decreases (*φ* > *φ*_*c*_), with increasing the *φ*. In the insulative regime, the dielectric constant (*ε*_*r*_) of the composites can be written as:^5^,^43^,^44^,^45^,^46^


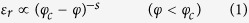


where *s* (>0) is a constant. Based on the experimental results, it is found that “*s*” can be as small as 0.17 and as high as 1.78^47^,^48^. From the data shown in Fig. 6(b), one would expect that the *φ*_*c*_ is somewhere between 0.4 to 0.5. Using Eq. (1) to fit the data (*φ* = 0 to *φ* = 0.4) shown in Fig. 6(b), it is obtained that *φ*_*c*_* = *0.47 and s* = *1.57. That is, if the composites are treated as CDCs, the composites with 50 vol% CCTO (i.e. *φ* > *φ*_*c*_) would be a conductor. However, the dielectric loss observed in the composites with 50 vol% CCTO is less than 0.35 at 100 Hz and 0.25 at 1 kHz, respectively. As will be discussed in next section, the composites with 50 vol.% CCTO are still insulative and its dielectric loss at room temperature is dominated by the dielectric response.

### Temperature Dependence of Dielectric Properties

**Figure 7:**
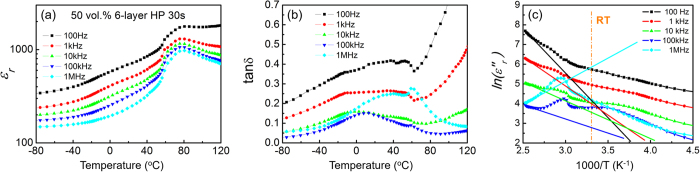
(**a**) Dielectric constant versus temperature, (**b**) loss versus temperature, (**c**) imaginary part of dielectric permittivity versus 1000/T, where the solid lines are the fitting results using Eqs (2) and (3), for the composites with 50 vol.% of CCTO prepared using 6 layers with a PCCP configuration for 30 s.

The temperature dependence of the dielectric properties was characterized as shown in Fig. 7(a,b), where the results from the composites with 50 vol% CCTO that exhibit the highest dielectric constant are presented. The composites exhibit a high dielectric constant with a low loss over a wide temperature range. For example, the dielectric constant at 100 Hz and 1 kHz is more than 1,000, which is significantly higher than the dielectric constant obtained in other DDCs and is similar to the dielectric constant observed in CDCs with a *φ* close to *φ*_*c*_. However, the composites reported have a much smaller loss (tanδ ~ 10^−1^) than the CDCs (tanδ ~ 10^0^ to 10^2^).

To study the nature of the dielectric loss observed in the composites, the imaginary part (*ε*″_*r*_* = ε*_*r*_ · tan*δ*) of the dielectric permittivity is plotted in Fig. 7(c). For a dielectric with a considerable electric conductivity, the measured *ε*″_*r*_ originates from: (1) real dielectric response (*ε*″_*r,real*_) such as dielectric relaxation processes and (2) electrical conductivity (*σ*). That is, the measured *ε*″_*r*_ can be written as:^49^


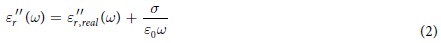


where *ω* is the angular frequency and *ε*_*0*_ ( = 8.85 × 10^−12^ F/m) is the dielectric permittivity of free space. It is well known that the temperature dependence of the *σ* can be written as:^5^


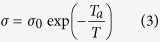


where *T* is the temperature in Kelvin, *σ*_*0*_ is the pre-exponential factor, and *T*_*a*_ is the equivalent temperature of the activation energy. Based on Eqs (2) and (3), one can get that if the measured *ε*″_*r*_ is dominated by the electrical conductivity, a linear relationship would be obtained between ln(*ε*″_*r*_) and *1/T* at one each frequency. Based on Eq. (3), the electrical conductivity increases with increasing temperature or the conductivity has a stronger contribution to the *ε*″_*r*_ at higher temperatures. Assuming that the measured *ε*″_*r*_ at high temperatures is dominated by the electrical conductivity, one can use Eq. (3) to fit the data shown in Fig. 7(c) for high temperatures. The fitting results are shown in Fig. 7(c) as the solid lines. Based on Eq. (2), the lower the frequency is, the stronger the contributor of the conductivity to the *ε*″_*r*_ is. That is, the conductivity has the strongest influence on the *ε*″_*r*_ at 100 Hz among all frequencies used. From Fig. 7(c), one can find for the results at 100 Hz that the measured *ε″*_*r*_ at room temperature is significantly higher than the solid line. Additionally, if the *ε*″_*r*_ is dominated by the electrical conductivity, the fitting results at different frequencies should have the same slope. As shown in Fig. 7(c), the fitting lines for different frequencies are different. For the results at 1 MHz, the slope of the fitting results even has a different sign comparing to that at 100 Hz (i.e. the measured *ε*″_*r*_ is dominated by the real dielectric response). All of this indicates that even at a temperature as high as 120 °C, the *ε*″_*r*_ is not dominated by the electric conductivity. Therefore, one can conclude that the measured *ε*″_*r*_ or the dielectric loss of the composites reported here, especially at low temperatures including room temperature, is mainly determined by the real dielectric response which includes the response from the dielectric process induced by the mixing.

**Figure 8:**
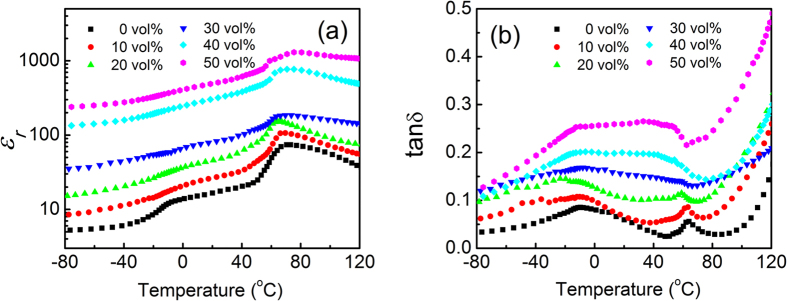
Temperature dependence of (**a**) dielectric constant and (**b**) loss at 1 kHz for the composites with different CCTO contents shown in Fig. 6(b).

As shown in Fig. 7(a,b), the dielectric constant of the composites exhibits a maximum peak at a temperature (*T*_*max*_) around 70 °C. To study the nature of this maximum peak, the temperature dependence of the dielectric properties is presented in Fig. 8 for the composites shown in Fig. 6(b). Clearly, the dielectric constant of the matrix shows a peak at a temperature around 70 °C that is its ferroelectric-to-paraelectric phase transition temperature^49^. Therefore, the fact that a dielectric-constant-maximum peak appears at the *T*_*max*_ for all composites is due to the phase transition in the polymer matrix. For the matrix, there is a step increase in the dielectric constant at temperatures around −10 °C, which is associated with its glass transition temperature (*T*_*g*_). From the results shown in Fig. 8(a), one can find that the composites exhibit a significantly weaker temperature dependence of the dielectric constant than for the polymer matrix at temperatures around *T*_*g*_ and *T*_*max*_. This reflects the changes in the matrix due to the appearance of CCTO. It is expected and supported by the X-ray diffraction data that both the size of the polymer crystals and the crystallinity in the polymer matrix decreases as *φ* increases since the CCTO particles limit the growth of the polymer crystals. For the polymer matrix used here, a lower crystallinity would result in a weaker peak for the dielectric constant at *T*_*max*_ and a smaller size of the polymer crystals has a lower ferroelectric-to-paraelectric phase transition temperature^50^. Therefore, as the *φ* increases, the temperature dependence of the dielectric constant around *T*_*max*_ would become weaker and the *T*_*max*_ should decrease, which are exactly what is observed in Fig. 8(a).

The reduction in the crystallinity and the size of the polymer crystals in the matrix with increasing *φ* is also confirmed by the temperature dependence of the dielectric loss, where a loss peak appears at the phase transition temperature. Regarding the *T*_*g*_, it is well known that as the *φ* of the composites increases the *T*_*g*_ of a polymer matrix decreases and broadens^11^,^51^, which would result in a weaker temperature dependence of the dielectric constant at temperatures around the *T*_*g*_. This is what is observed in Fig. 8(a). The loss shown in Fig. 8(b) also confirms the change in the *T*_*g*_. At temperatures higher than 80 °C, the loss increases with the temperature as shown in Fig. 8(b), which is due to the increase in the electric conductivity as described above. In other words, at temperatures below 80 °C, the loss of the composites is mainly determined by the real dielectric responses.

## Conclusions

In conclusion, it is experimentally demonstrated that the dielectric constant of a polymer-based 0–3 composite can be significantly enhanced by combining solution-cast and hot-pressing processes. More interestingly, it is found that the stack configuration of the as-cast films used in the hot-pressing process plays an important role on the dielectric enhancement. By using a proper hot-pressing condition, composites with an ultra-high dielectric constant are developed. For example, the dielectric constant of the CCTO-P(VDF-TrFE) composites can exceed 1,000, which is significantly higher than the dielectric constant observed in other DDCs. This ultra-high dielectric constant is comparable to the dielectric constant obtained in the CDCs, but the composites developed here have a much smaller dielectric loss (10^−1^) compared to the conductor-dielectric composites (10^0^–10^2^). It is concluded that besides eliminating porosity the redistribution of the filler particles in the matrix during the hot-press process plays an important role on the observed high dielectric constant. The experimental results indicate that the composite with a uniform microstructure (i.e. the distribution of the filler particles in a matrix) exhibits a higher dielectric constant. It is also found that the dielectric constant of the composites exhibit a weaker temperature dependence at temperatures around *T*_*g*_ and *T*_*max*_.

## Methods

### Synthesis of CaCu_3_Ti_4_O_12_ (CCTO) ceramics

The CaCu_3_Ti_4_O_12_ (CCTO) ceramics were prepared by using a traditional powder processing method. High-purity metal oxide powders of Calcium Carbonate (CaCO_3_, 99.5%, Alfa Aesar), Copper Oxide (CuO, 99.7%, Alfa Aesar) and Titanium Dioxide (TiO_2_, 99.8%, Alfa Aesar) were used to prepare the ceramics. The ceramics were calcined at 900 °C and sintered at 1075 °C for 72 h^52^. The dielectric behavior of the ceramics at room temperature is shown in Fig. 1. The ceramic pellets were milled to powders to prepare the micro-sized CCTO particles, which were used as the fillers for the composites reported here.

### Preparation of CCTO-P(VDF-TrFE) composites

P(VDF-TrFE) 55/45 mol.% copolymer (i.e. polymer matrix) was first dissolved in dimethyl formamide (DMF) using magnetic stirring for 8 h at room temperature. The micro-sized CCTO particles were then added to the solution and stirred for 8 h, which was followed with a 20 min sonication. The final CCTO-P(VDF-TrFE) suspension/solution was cast on a pre-heated glass plate at 70 °C for 8 h. The CCTO-P(VDF-TrFE) composites with 0, 10, 20, 30, 40, 50, and 60 vol.% of CCTO were prepared. The thickness of as-cast film is around 50–80 μm. The as-cast film was annealed at 125 °C for 8 h to form the solution-cast composites.

A hot-pressing process was used to prepare the final composites using the as-cast film as the starting materials. The as-cast films with the same compositions were stacked together to form a multilayer stack with two different configurations. The multilayer stack was hot pressed at 200 °C for different times: 10, 20 and 30 s, respectively. Finally, the hot-pressed samples were annealed at 125 °C for 8 h by placing it between two glass plates.

### Characterization

The morphology and the uniformity of the composite films were examined using a JEOL JSM 7000F FE-SEM. The samples used in SEM were prepared by breaking composite in liquid nitrogen and the cross-sections were used in the SEM observations. The structure of the composites was characterized with D8 Discover X-ray Diffractometer. For the characterization of the dielectric properties of the composites, the samples were sputtered with gold in a circular shape with a diameter of 3 mm on both surfaces as electrodes. An Agilent 4294A Impedance Analyzer was used to characterize the capacitance and loss using Cp ~ D function. The dielectric constant was calculated from the capacitance using the parallel plate mode. The relative error of the dielectric properties was calculated based on the measured results of 3 to 5 samples of a composite. The dielectric properties of the composites at room temperature were characterized over a frequency range from 100 Hz to 1 MHz. The temperature dependence of the dielectric properties was characterized at 100 Hz, 1 kHz, 10 kHz, 100 kHz and 1 MHz over a temperature range from −80 °C to 120 °C with a heating rate of 3 °C/min.

## Additional Information

**How to cite this article**: Zhang, L. *et al*. Process and Microstructure to Achieve Ultra-high Dielectric Constant in Ceramic-Polymer Composites. *Sci. Rep.* **6**, 35763; doi: 10.1038/srep35763 (2016).

## Figures and Tables

**Table 1:** Summary of dielectric constant (at 100 Hz) for the composites prepared using different hot pressing processes: hot pressing time and number of layers used in the hot pressing stack.

Hot pressing time	Layer number	CaCu_3_Ti_4_O_12_ Concentration (vol.%)					
		10 vol.%	20 vol.%	30 vol.%	40 vol.%	50 vol.%	60 vol.%
as-cast	1 layer	27.5	38.1	49.6	136.1	77.7	—
10 s	2 layer	29.3	48.1	58.6	251.0	454.1	72.8
	4 layer	28.6	39.6	60.9	107.0	116.8	112.2
	6 layer	28.7	47.9	103.9	126.0	256.1	305.1
20 s	2 layer	26.6	36.7	51.9	446	227	245
	4 layer	27.9	42.7	66.0	370.0	236.1	202.2
	6 layer	27.1	62.7	65.9	104.0	462.3	307.8
30 s	2 layer	24.6	29.6	61.3	292.1	253.1	213.2
	4 layer	27.4	42.0	70.5	133.1	381.2	197.9
	6 layer	28.3	49.3	51.6	108.2	758.0	352.2
